# Ⅰ期非小细胞肺癌达芬奇机器人手术的疗效分析

**DOI:** 10.3779/j.issn.1009-3419.2018.11.07

**Published:** 2018-11-20

**Authors:** 星池 刘, 世广 许, 博 刘, 惟 徐, 仁泉 丁, 通 王, 博 李, 希龙 王, 琼 吴, 洪 滕, 述民 王

**Affiliations:** 110016 沈阳，沈阳军区总医院胸外科 Department of Thoracic Surgery, General Hospital of Shenyang Military Command, Shenyang 110016, China

**Keywords:** 达芬奇机器人手术系统, 肺肿瘤, 生存状况, 无进展生存状况, 生存分析, Da Vinci S Surgical System, Lung neoplasms, Overall survival, Disease free survival, Survival analysis

## Abstract

**背景与目的:**

达芬奇机器人手术系统在胸外科的应用日益广泛，本研究旨在探讨经达芬奇机器人手术治疗Ⅰ期非小细胞肺癌（non-small cell lung cancer, NSCLC）患者的疗效。

**方法:**

回顾2012年1月-2017年12月于我科行手术治疗的Ⅰ期NSCLC患者347例，依据手术方式分为机器人（robot-assisted thoracic surgery, RATS）组134例及腔镜（video-assisted thoracic surgery, VATS）组213例。比较两组患者围术期一般指标（术中出血量、术后引流量、术后带管时间、术后住院时间、淋巴结清扫状况），分析患者生存状况（overall survival, OS）、无进展生存状况（disease free survival, DFS）及相关影响因子。

**结果:**

机器人组与腔镜组术中出血量[(49±39) mL *vs* (202±239) mL]、术后引流量[Day 1: (248±123) mL *vs* (350±213) mL; Day 2: (288±189) mL *vs* (338±189) mL]比较，机器人组均少于腔镜组（*P* < 0.05）；术后带管时间[(10±5) d *vs* (11±8) d]及住院时间[(13±6) d *vs* (14±9) d]两组患者无明显差异（*P* > 0.05）。机器人组与腔镜组的淋巴结清扫组数[（5±2）组*vs*（4±2）组]及淋巴清扫数量[（18±9）枚*vs*（11±8）枚]比较，机器人组均优于腔镜组（*P* < 0.05）。机器人组与腔镜组生存状况比较[1年生存率：97.3% *vs* 96%、3年生存率：89.8% *vs* 83.1%、5年生存率：87.5 % *vs* 70.3%，平均生存时间（month）：61 *vs* 59]，两组无统计学差异（*P* > 0.05）。无进展生存状况：机器人组与腔镜组比较[1年无进展生存率：93.7% *vs* 91.3%、3年无进展生存率：87.7% *vs* 68.4%、5年无进展生存率：87.7% *vs* 52.5%，平均无进展生存时间（month）：61 *vs* 50]，机器人组明显优于腔镜组（*P* < 0.05）。单因素分析显示，淋巴结清扫数量是患者生存状况的影响因子；肿瘤直径、手术方式、淋巴结清扫组数、淋巴结清扫数量为患者无进展生存状况的影响因子。多因素分析显示生存状况无独立影响因子，肿瘤直径及手术方式为无进展生存状况的独立影响因子。

**结论:**

达芬奇机器人Ⅰ期非小细胞肺癌患者术后生存状况与腔镜手术无差异，但无进展生存状况优于腔镜手术；达芬奇机器人手术淋巴结清扫更彻底，同时术中出血量更少。

## Abstract (en)

**Background and objective:**

Da Vinci robotic surgery system is widely used in department of thoracic surgery. The aim of this study is to investigate the treatment outcome of stage Ⅰ non-small cell lung cancer (NSCLC) via da Vinci Surgical System.

**Methods:**

Clinical date of 347 stage Ⅰ NSCLC patients, who underwent lobectomy and systematic node dissection from Jan. 2012 to Dec. 2017, were reviewed. 134 patients underwent robot-assisted thoracic surgery (RATS) and 213 patients underwent video-assisted thoracic surgery (VATS). To compare perioperative outcome (blood lose, postoperative drainage, drainage time, postoperative hospital stay, number of the LN dissection) and analyze overall survival (OS), disease free survival (DFS) of the two groups and prognostic factors.

**Results:**

The RATS group got less blood lose [(49±39 mL for RATS *vs* (202±239) mL for VATS, *P* < 0.05] and postoperative drainage [Day 1: (248±123) mL for RATS *vs* (350±213) mL for VATS; Day 2: (288±189) mL for RATS *vs* (338±189) mL for VATS, *P* < 0.05]. There were no significant difference for drainage time (10±5 for RATS *vs* 11±8 for VATS, *P* < 0.05) and postoperative hospital stay (13±6 for RATS *vs* 14±9 for VATS, *P* < 0.05) between the two groups. The RATS group harvested a more number of mean stations (5±2 for RATS *vs* 4±2 for VATS) and amounts (18±9 for RATS *vs* 11±8 for VATS) of the lymph nodes, *P* < 0.05. There was no statistically significant difference of OS between RATS and VATS group [1-year OS: 97.3% *vs* 96%; 3-year OS: 89.8% *vs* 83.1%; 5-year OS: 87.5 % *vs* 70.3%; overall survival time (mean): 61 months *vs* 59 months, *P* > 0.05]; corresponding there had a statistically significant difference of DFS between the two groups [1-year DFS: 93.7% *vs* 91.3%; 3-year DFS: 87.7% *vs* 68.4%; 5-year DFS: 87.7% *vs* 52.5%; disease free survival time (mean): 61 months *vs* 50 months, *P* < 0.05]. The univariate analysis found that the amounts of the lymph nodes dissection was the prognostic factor for OS and tumor diameter, surgical approach, stations and amounts of the lymph nodes dissection were respectively the prognostic factors for DFS. However, multivariate analysis found that there was not independently factors for OS, but the tumor diameter and surgical approach were independently associated with DFS.

**Conclusion:**

There was no significant difference about OS between the two groups, but the RATS got better DFS. RATS got more number of the LN dissection and less blood lose.

达芬奇机器人手术系统在胸外科的应用从早期的质疑、尝试，已逐步过渡到为人熟知，现今广泛应用于胸外科手术中。但在早期非小细胞肺癌的治疗中，与技术成熟完善的腔镜手术相比，能否有一定优势仍未有定论。因此我们回顾Ⅰ期非小细胞肺癌患者同期行达芬奇机器人手术和腔镜手术的术后临床数据，分析比较两者的治疗效果及影响因子。

## 1 资料与方法

### 1.1 一般资料

本组资料纳入2012年1月-2017年12月于我科行肺癌根治术且术后病理回报为非小细胞肺癌、肿瘤-淋巴结-转移（tumor-node-metastasis, TNM）（第8版）分期Ⅰ期患者347例，其中行机器人手术患者134例，行胸腔手术患者213例。两组患者术后病理腺癌较多，腔镜组鳞癌较机器人组略多，其余一般临床资料无明显差异，见表 1。

**1:** 一般临床状况（*n*=347） Clinical characteristics of patients (*n*=347)

Group	Age (yr)	Gender			Histologic type				Location				
		Male	Female		SQCC	ADC	Others		RUL	RML	RLL	LUL	LLL
RATS	62.14±8.6	67	67		9	104	21		46	7	28	25	28
VATS	61.3±8.0	118	95		43	137	33		57	7	53	64	32
*P*	0.365	0.326			0.002				0.069				
RUL: right upper lobe; RML: right middle lobe; RLL: right lower lobe; LUL: left upper lobe; LLL: left lower lobe; SQCC: squamous cell carcinoma; ADC: adenocarcinoma.													

### 1.2 手术方法

胸腔镜组：患者取健侧卧位，于腋中线第7或8肋间打1.5 cm作进镜口，腋前线第4或5肋间切3 cm-5 cm作操作孔。机器人组：取健侧折刀位，于腋后线第7或8肋间切1.5 cm小口作进镜口，腋前线第5肋间和肩胛线第7或8肋间分别切1 cm小口作操作臂口，腋中线第7肋间切3 cm小口作辅助口。如果术前病理或术中快速冰冻病理检查提示恶性，则均行肺叶切除加系统淋巴结清扫，淋巴结清扫范围右侧为2组-4组、7组-12组淋巴结，左侧为4组、5组、6组、7组-12组淋巴结。

### 1.3 随访方法

随访起点时间为手术后1个月，随访终点时间为2017年12月。生存时间（overall survival, OS）为术后1个月至因肿瘤死亡时间、无进展生存时间（disease free survival, DFS）为术后1个月至肿瘤复发时间。术后两年内每3个月行电话随访，之后每半年随访一次直至病故。至随访终点时间（2017年12月）前出现死亡或复发记为结局事件，中途失访或至随访终点时间仍未出现结局事件纳入删失值，最终计算累积生存率。

### 1.4 统计学处理

采用SPSS 23.0统计软件。连续性变量和分类变量分别以秩和检验及卡方检验比较。采用*Kaplan-Meier*法计算生存率并绘制生存曲线。生存因子单因素分析选用*Log-rank*检验，部分连续性变量采用*Cox*回归；多因素分析采用*Cox*回归模型，以*P* < 0.05为差异有统计学意义。

## 2 结果

### 2.1 围术期一般指标

术中出血量、术后两天引流量状况上机器人组少于腔镜组（*P* < 0.05），在术后胸引管留置时间及术后住院时间上两组患者无统计学差异（*P* > 0.05）。术中淋巴结清扫组数及淋巴结清扫总量上比较，机器人组优于腔镜组（*P* < 0.05），见表 2。

**2:** 围术期结果（Mean±SD） Perioperative outcome (Mean±SD)

Parameter	RATS	VATS	*P*
Blood lose (mL)	49±39	202±239	0.000
Postoperative drainage (mL)			
Day 1	248±123	350±213	0.000
Day 2	288±189	338±189	0.002
Drainage time (d)	10±5	11±8	0.119
Postoperative hospital stay (d)	13±6	14±9	0.520
Station of the LN dissection	5±2	4±2	0.000
Number of the LN dissection	18±9	11±8	0.000
RATS: robotic-assisted thoracic surgery; VATS: video-assisted thoracic surgery; LN: lymph node.			

#### 2.2.1 术后生存状况单因素分析

机器人组及腔镜组术后生存状况无统计学差异（*P* > 0.05）；单因素分析显示淋巴结清扫数量为术后生存状况的预后影响因子，见表 3、图 1。

**3:** 术后生存状况的单因素分析 Univariate analysis of prognostic factors for overall survival time

Parameter	Cases	Overall survival rate (%)				Overall survival time (mo)			*P*
		1-year	3-year	5-year		Mean	SE	95%CI	
Age (continues)^a^	31	-	-	-		-	-	-	0.296
Gender									0.639
Male	18	94.9	85.3	79.1		60	2	57-64	
Female	13	98.4	86.9	65.9		60	3	55-65	
Smoking									0.097
Never	11	98.4	90.1	73.6		62	2	58-66	
Ever	20	99.4	96.8	75.6		59	2	55-63	
Histological type									0.323
SQCC	7	93.9	77.9	77.9		57	4	50-65	
ADC	18	97.3	89.6	75.4		61	2	58-65	
Others	6	95.6	78.9	-		49	3	43-54	
Cell differentiation									0.823
Well	1	100.0	75.0	75.0		51	4	43-59	
Moderately	15	96.8	89.9	74.6		61	2	58-65	
Poorly/undifferentiated	11	95.7	83.8	80.3		59	2	55-64	
Unknown	4	96.3	78.8	-		48	3	42-55	
Tumor diameter (continues)^a^	31								0.151
Approach									0.260
RATS	10	97.3	89.8	87.5		61	2	58-65	
VATS	21	96.0	83.1	70.3		59	2	55-63	
Station of the LN dissection									0.150
≤4	21	96.1	83.9	70.5		59	2	55-63	
> 4	10	97.1	89.0	86.2		62	2	59-66	
Number of the LN dissection									0.030
≤12	22	95.2	80.4	67.3		57	2	53-62	
> 12	9	98.1	91.9	89.2		64	2	61-67	
^a^*Cox* regression analysis.									

**1:**
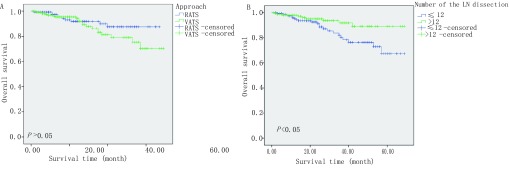
单因素分析相关的总生存曲线。A：不同手术方式的总生存曲线，刻度标记代表删失数据；B：不同淋巴结清扫数量的总生存曲线，刻度标记代表删失数据。 Overall survival curves related to univariate analysis. A: Overall survival curves for different approach, tick marks represent censored data; B: Overall survival curves for different number of the LN dissection, tick marks represent censored data.

#### 2.2.2 术后生存状况多因素分析

将各变量带入*Cox*回归模型显示，各变量均不是术后生存状况独立性风险因素。

#### 2.2.3 术后无进展生存状况单因素分析

机器人组及腔镜组术后无进展生存状况比较，机器人组明显优于腔镜组（*P* < 0.05）；单因素分析显示肿瘤直径、手术方式、淋巴结清扫组数、淋巴结清扫数量为术后无进展生存状况的预后影响因子，见表 4、图 2。

**4:** 术后无进展生存状况的单因素分析 Univariate analysis of prognostic factors for disease free survival time

Parameter	Cases	Disease free survival rate (%)				Disease free survival time (mo)			*P*
		1-year	3-year	5-year		Mean	SE	95%CI	
Age (continues)^a^	54	-	-	-		-	-	-	0.075
Gender									0.983
Male	29	91.7	79.1	66.1		55	2	51-60	
Female	25	93.0	72.7	66.1		55	3	50-60	
Smoking									0.723
Never	26	92.2	75.3	68.9		56	2	51-60	
Ever	28	92.3	77.1	63.4		55	2	50-59	
Histological type									0.451
SQCC	11	88.7	73.2	56.3		50	4	43-59	
ADC	34	93.8	78.6	68.2		56	2	53-60	
Others	9	89.3	69.2	-		45	3	38-51	
Cell differentiation									0.271
Well	1	100.0	75.0	-		51	4	43-59	
Moderately	26	92.9	82.3	64.4		57	2	52-61	
Poorly/undifferentiated	19	91.9	72.6	69.8		53	3	48-59	
Unknown	8	87.0	61.3	-		41	4	33-50	
Tumor diameter (continues)^a^	54	-	-	-		-	-	-	0.002
Approach									0.001
RATS	11	93.7	87.7	87.7		61	2	57-65	
VATS	43	91.3	68.4	52.5		50	2	45-55	
Station of the LN dissection									0.008
≤4	39	89.0	70.2	57.2		51	2	47-56	
> 4	15	96.4	84.7	80.1		60	2	56-63	
Number of the LN dissection									0.041
≤12	35	90.3	70.8	57.8		52	2	47-57	
> 12	19	94.5	82.5	78.2		59	2	55-63	
^a^*Cox* regression analysis.									

**2:**
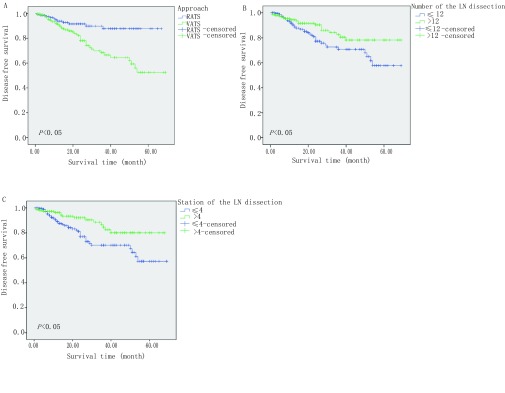
单因素分析相关的无进展生存曲线。A：不同手术方式的无进展生存曲线，刻度标记代表删失数；B：不同淋巴结清扫数量的无进展生存曲线，刻度标记代表删失数据；C：不同淋巴清扫组数的无进展生存曲线，刻度标记代表删失数据。 Disease free survival curves related to univariate analysis. A: Disease free survival curves for different approach, tick marks represent censored data; B: Disease free survival curves for different number of the LN dissection, tick marks represent censored data; C: Disease free survival curves for different number of the LN dissection, tick marks represent censored data.

#### 2.2.4 术后无进展生存状况多因素分析

将各变量带入*Cox*回归模型显示，肿瘤直径及手术方式分别为术后无进展生存状况的独立风险因素，见表 5。

**5:** 术后无进展生存状况的多因素分析 Multivariate analysis of prognostic factors for disease free survival time

Parameter	Multivariate analysis		
	HR	95%CI	*P*
Tumor diameter (continues)	1.548	1.1-2.2	0.010
Approach			
RATS	Reference		
VATS	2.610	1.3-5.1	0.005

## 3 讨论

近几年来，肺癌一直是发病率较高的常见恶性肿瘤，其发病率在男性人群中位于第一位、女性人群位于第二位。在肺癌组织学分型上，非小细胞肺癌占大多数，手术治疗在当下仍旧为非小细胞肺癌的首选治疗方案。经多学科共识，快速康复外科（enhanced recovery after surgery, ERAS）成为如今外科治疗的发展趋势之一，如何在安全有效的手术治疗基础上，达成更小的创伤、更快的术后恢复是当下焦点问题^[1]^。随着多年临床实践的经验累积，腔镜手术在胸外科的应用广泛、技术成熟。早先大数据显示：在早期肺癌的手术治疗中，腔镜手术的治疗效果与传统开胸手术相当，安全可行、并发症较少，而生存期甚至更好^[2, 3]^。与此同时患者的创伤却大大减小，术后疼痛明显减轻、引流管留置时间及术后住院时间短，恢复更快^[4-7]^。达芬奇机器人手术作为胸外科微创手术的另一选择，其具有独到优势，在胸外科的应用从早期的质疑、尝试，已逐步过渡到为人熟知，且广泛应用于胸外科手术当中^[8-11]^。首先其成像系统可提供最高达15倍的图像放大倍数，同时控制台提供一个3D图像，这与目前3D胸腔镜要求术者带成像眼镜不同，达芬奇机器人双目视频镜头采集图形后，通过控制台提供一个裸眼3D图像，故可以给术者提供更加清晰的手术视野。器械臂末端关节具有7个自由度及双轴向的转腕功能，利于在狭小空间操作；另外其通过动态过滤器可消除人手的抖动，故此达芬奇机器人在精细分离、缝合等方面有着得天独厚的优势，从而使患者的术中创伤更小^[12, 13]^。从我们的临床数据观察，在Ⅰ期非小细胞肺癌手术中，达芬奇机器人手术平均出血量（49±39）mL、腔镜手术平均出血量（202±239）mL，机器人手术中出血量明显少于腔镜手术（*P* < 0.05）。在术后引流量方面，机器人组术后第1天、第2天引流量分别为（248±123）mL、（288±189）mL；腔镜组第1天、第2天引流量分别为（350±213）mL、（338±189）mL，机器人组的术后48 h引流量亦明显少于腔镜组（*P* < 0.05）。

非小细胞肺癌纵隔淋巴结转移是影响预后的主要因素。通常胸腔内淋巴结转移遵循特定规律，多数是由近及远、由肺段肺叶经肺门向纵隔转移，也因此肺癌淋巴结转移具有多发性，即同时多组淋巴结转移。从在解剖学上讲，肺叶胸膜下淋巴管是可直接回流至纵隔淋巴结的，于是便出现了肺癌淋结的跳跃性转移^[14, 15]^。术中淋巴结清扫极为重要，其不仅可以清除已有转移的淋巴结，同时还切断了经淋巴结的转移途径^[16, 17]^。目前早期非小细胞肺癌淋巴清扫方式一直较有争议，一类是系统性淋巴结采样（systematic sampling），另一类是系统性淋巴结清扫（systematic node dissection, SND）^[18, 19]^。在淋巴清扫方式的选择上，我们一直坚持系统性淋巴结清扫。早先有研究认为，对于Ⅰ期非小细胞肺癌，系统性淋巴结清扫创伤较大、增加术中出血量和术后引流量、同时可导致局部免疫功能下降，对于患者的生存期无明显延长^[20]^。从我们的数据观察，机器人组术中出血量平均为（49±39）mL、腔镜组平均出血量（202±239）mL，淋巴结清扫数量增加在所难免造成术中渗出较多；在术后引流时间，机器人及腔镜组引流管留置时间分别为（10±5）d、（11±8）d，两组引流管留置时间虽无统计学差异（*P* > 0.05），但时间确实略有偏长。回顾分析原因，一是早先我们对于拔管指征较为保守，连续两天胸引量小于100 mL，颜色淡黄后予以拔管；其次可能确实为淋巴结清扫数量略多、范围较广所致。虽然我们要面对系统淋巴结清扫时淋巴清扫数量增加可能造成术中出血量、术后引流量增加及引流管留置时间延长等负面状况，但对于现今尚无良好的办法在术前甚至术中明确有无纵隔淋巴结转移及转移范围，Ⅰ期非小细胞肺癌行肺叶切除加系统性淋巴结清扫，确实能使患者总生存率和无进展生存率得以提高^[21, 22]^。系统性淋巴结清扫规范化的淋巴结清扫组数及清扫数量，不仅将可能隐匿性转移的淋巴结清除，而且还可提供更多的标本量，降低病理检查漏诊率，提高病理的准确性，得出更精准的TNM分期，为术后指导治疗提供可靠依据，从而使患者获得更好的生存获益^[23, 24]^。我们的研究数据亦证实，淋巴结清扫数量 > 12枚的术后OS结果要优于淋巴结清扫数量≤12枚的OS状况，有明显统计学差异（*P* < 0.05）；与此同时，淋巴结清扫组数 > 4组、淋巴清扫数量 > 12枚的术后DFS结果亦优于淋巴结清扫组数≤4组、淋巴清扫数量≤12枚的DFS状况，有明显统计学差异（*P* < 0.05）。单因素分析显示，淋巴结清扫数量为术后OS状况的影响因子；对于术后DFS状况，淋巴结清扫组数、淋巴结清扫数量均为其影响因子。

综上所述，与腔镜手术相比较，达芬奇机器人手术在Ⅰ期非小细胞肺癌的治疗中，淋巴结清扫更加彻底（RATS: 18±9 *vs* VATS: 11±8, *P* < 0.05）的同时能减少术中出血量及术后引流量，但亦无法避免系统性淋巴结清扫所致的引流管留置时间较长问题；从术后DFS状况来看，单因素分析及*Cox*多因素分析均显示：手术方式为术后DFS状况的影响因子，达芬奇机器人手术对于术后DFS状况有明显优势（*P* < 0.05）；从术后OS状况分析，达芬奇机器人手术与腔镜手术无明显差异（*P* > 0.05）。故单从术后OS状况考虑，腔镜手术与达芬奇机器人手术对于Ⅰ期非小细胞肺癌的治疗效果不分伯仲，但DFS时间的延长势必影响患者术后生存质量，也因此随着样本量的增大、多中心结果的汇总以及随访时间的延长，达芬奇机器人手术在早期非小细胞肺癌的长远效果让人期待。本研究结果为我单中心数据得出，亦有些许不足之处。淋巴结清扫数量虽然为术后OS、DFS的预后影响因子，但淋巴结清扫的程度如何掌控，以便在术后受益及副损伤间得到最佳阈值，仍需进一步研究。本研究资料腔镜组术中出血量偏多，分析其潜在原因，一可能因为过多清扫纵隔淋巴结，其二由于入组腔镜手术患者中有4例因术中粘连渗血较多（平均1, 225 mL），另有1例左肺上叶术中肺动脉干出血（2, 500 mL）所致。另外入组患者的术后病理分型存在差异，虽然数据的单因素分析显示：术后病理分型不是OS、DFS状况的影响因子，但不同病理分型术后治疗策略的差异亦可能对术后生存状况产生偏倚。
